# Serum Reference Values for Leptin in Healthy Infants

**DOI:** 10.1371/journal.pone.0113024

**Published:** 2014-11-21

**Authors:** Francesco Savino, Lorenza Rossi, Stefania Benetti, Elisa Petrucci, Miriam Sorrenti, Leandra Silvestro

**Affiliations:** Department of Paediatrics, University of Turin, Regina Margherita Children's Hospital, Turin, Italy; University of Cordoba, Spain

## Abstract

**Objective:**

Reports on leptin concentrations in pediatric populations lack reference values for infants in the first months of life. Our study was conducted on healthy full-term infants between 2002 and 2012 to determine serum leptin reference values in subjects less than 18 months old.

**Methods:**

Routine outpatient blood tests for serum leptin were performed on 317 infants using a radioimmunoassay method. The median and 10^th^–90^th^ percentiles were calculated to obtain reference values using quantile regression. Values established in this study were compared with another independent cohort of 110 infants.

**Results:**

The median (IQR) serum leptin concentration in the infants was 2.37 (3.26) ng/ml (*n* = 317). The median leptin concentration was 2.81 (3.49) ng/ml (*n* = 202) in infants younger than 6 months of age, 1.44 (2.27) ng/ml (*n* = 59) in infants between 6–12 months of age and 1.77 (2.05) ng/ml (*n* = 56) in infants between 12–18 months of age. We obtained leptin reference values based on age by estimating the lower and upper percentiles. In the entire cohort, the median (IQR) leptin concentration was 2.22 (3.11) ng/ml in males (*n* = 168) and 2.60 (3.32) ng/ml in females (*n* = 149). According to the type of feeding median serum leptin concentration was higher in breast-fed infants (n = 188) than in formula-fed infants (n = 129) (2.63 (3.34) ng/ml vs. 2.12 (2.77) ng/ml; p<0.05).

**Conclusions:**

Our data revealed no gender difference in leptin concentration in early infancy. After 6 months of life, leptin concentrations decreased slightly. We used a large cohort to confirm that breast-fed infants had significantly higher serum leptin levels than formula-fed infants during the first 6 months of life, although this difference disappeared later in life. In this study, we defined the leptin reference range in healthy infants in the first 18 months of life according to the Clinical and Laboratory Standards Institute (CLSI).

## Introduction

Leptin, a 167-amino-acid hormone that belongs to the adipocytokine family, was discovered in 1994. Leptin is encoded by the obesity (ob) gene on human chromosome 7q31.3 [1]. An autosomal recessive mutation in ob results in profound hyperphagia, obesity, reduced energy expenditure, hyperinsulinemia and insulin resistance [2]. Leptin is predominantly produced by white adipose tissue in an amount proportional to the body fat mass and, to a lesser extent, by the gastrointestinal tract, skeletal muscle, human placenta, mammary gland and foetal tissues [3], [4]. The main function of leptin is to inform the hypothalamic structures regarding energy deposits and to help regulate energy balance; leptin exerts an anorexigenic effect and increases energy expenditure [5]. However, during early infancy, leptin does not appear to play an anorexigenic role; leptin maintains an increased appetite in infants to promote their survival during the period in which they lack feeding independence [6].

Leptin also exert various peripheral functions, with pleiotropic effects on the physiology and pathophysiology of energy homeostasis, endocrinology and metabolism [7]. Evidence shows that serum leptin concentrations reflect body fat mass during foetal life, infancy, childhood and adulthood [8], [9]. Leptin has been detected in the foetus during the second trimester of pregnancy, and the levels of this hormone gradually increase with the development of foetal adipose tissue [10]. During periods of energy balance (weight maintenance), leptin concentrations in the blood reflect total body fat in both humans and mice. However, during periods of energy imbalance (gain or loss of weight), leptin concentrations indicate the direction of the imbalance. Therefore, leptin acts not only as an “adipostat” by signalling information about the amount of body fat to the brain but also as a sensor of energy imbalance [11].

It is conceivable that serum leptin concentrations in breastfed (BF) infants are related to adipose tissue production during the first months of life and to the leptin content of human milk. Leptin concentrations in breast milk positively correlate with maternal circulating leptin levels, BMI, and adiposity [12]. Data in the literature have described the changes that occur in leptin levels during intrauterine life, childhood and adulthood. However, large gaps remain in our knowledge of normal leptin levels during various periods of life, and a complete database of reference values is not yet available.

Reference intervals are essential for the interpretation of clinical laboratory tests and for the care of patients with signs or a family history of endocrine disorders or metabolic diseases. The aim of the present study was to establish and validate reference ranges of serum leptin concentrations in healthy Caucasian infants aged up to 18 months for use in clinical practice.

## Methods

### Reference Cohort

We enrolled AGA healthy term infants who were admitted to the Department of Paediatrics of the University of Turin, Regina Margherita Children's Hospital, between 2002 and 2012. The infants underwent blood tests during routine outpatient examinations. The inclusion criteria were the following: age <18 months; gestational age between 37 and 42 weeks; birth weight between 2500 and 4000 g; Apgar score >7 at 5 minutes; and exclusively BF or exclusively FF for at least 6 months. Following the initial 6-month period, all of the infants were fed based on the same complementary food scheme. The exclusion criteria included neonatal disease, fever, chronic illness or pathology indicating compromised growth.

At enrolment, the infants were weighed naked prior to feeding (electronic integrating scale, SECA, model 757, Vogel & Halke, Hamburg, Germany), the crown-to-heel length was measured (Harpenden Stadiometer, Holtain, Ltd, Crosswell, Crymych, Pembs., UK), the BMI was calculated (ratio of body weight (kg) to the square of length (m2)), and serum leptin levels were measured. The baseline characteristics of the infants at recruitment are summarized in Table 1.

**Table 1:** Baseline characteristics of infants in the reference cohort during recruitment.

Characteristics	Mean	Standard Deviation
Infant age (days)	185.09	160.36
Weight (kg)	6459.39	2127.02
Length (cm)	63.34	8.78
BMI (kg/m^2^)	15.71	2.18
Head Circumference (cm)	41.14	3.81

The study protocol was approved by the local Ethical Committee at Ospedale Mauriziano - Ospedale Infantile Regina Margherita - S. Anna Torino, and the infants' parents provided written consent to participate in the study.

### Validation Cohort

Between January 2013 and June 2014, healthy term infants visiting the Department of Paediatrics were screened for enrolment in the study. The exclusion criteria for these patients were identical to those used in the original study. The parents of 146 children who were eligible for the study were invited to participate in the study. After providing informed consent, the parents were asked to fill out screening questionnaires on health, learning and behaviour. Thirty-six of the invited children did not participate in this part of the study. A cohort of 110 infants (78 aged <6 months, 17 aged 6–12 months, 15 aged 12–18 months) was used for validation purposes.

### Sample collection and analysis

Venous blood samples were collected from the infants at 8 a.m., which coincided with the routine clinical blood sampling to minimise the disturbance to the infants. The samples were collected following a 3-hour fasting period. To determine the leptin concentration, serum was separated by centrifugation at 4000 rpm for 10 minutes and stored at −20°C. Immunoreactive leptin was measured using a commercially available radioimmunoassay (RIA) kit (LEP R-40, Human-Leptin-RIA-Sensitive, Mediagnost, Reutlingen, Germany) with a sensitivity of 0.04 ng/ml (0.01 ng/ml with the procedure for increased sensitivity). The intra-assay variation was less than 5%, and the inter-assay variation did not exceed 7.6% One blood sample was taken from each infant (n = 317). Each sample was divided into two serum samples containing different concentrations of leptin; leptin concentrations were measured 10 times in eight separate assays, and the final value was the median value of the 10 measurements.

### Statistical analysis

All the statistical analyses were conducted using SPSS software (version 19.0, SPSS, Inc., Chicago, IL). The normality of the distribution of the data was assessed with the Kolmogorov–Smirnov test. Continuous variables were expressed as the means ± standard deviations or medians and interquartile ranges (25th and 75th percentiles). The median and the 10^th^ and 90th percentiles were calculated to obtain reference values using quantile regression. Independent sample t-tests were used to compare the characteristics of the patients in the reference and validation cohorts for continuous data. Data that were not normally distributed (serum leptin levels in different groups) were analysed with the Mann–Whitney U test. Correlations are expressed by the Spearman correlation coefficient.

Statistical significance was set at p<0.05.

### Missing Data

No data were missing in either the reference cohort or the validation cohort.

## Results and Discussion

The median (IQR) serum leptin concentration in infants (*n* = 317) was 2.37 (3.26) ng/ml. Serum leptin values categorised by the infants' age, gender and type of feeding (breast vs. formula) are shown in Table 2.

**Table 2:** Serum leptin values according to infant's age, gender and type of feeding.

Groups	Serum leptin value (ng/ml)	*p*- value^a^
**Gender**		
Subjects aged <6 months		
Males *n* = 111	2.56 (3.2)	0.28
Females *n* = 91	3.36 (3.86)	
Subjects aged 6–12 months		
Males *n* = 29	1.37 (2.26)	0.36
Females *n* = 30	1.66 (2.54)	
Subjects aged 12–18 months		
Males *n* = 28	1.42 (2.15)	0.16
Females *n* = 28	2.2 (1.22)	
**Type of feeding**		
Subjects aged <6 months		
Breastfed *n* = 121	3.37 (3.56)	0.031
Formula fed *n* = 81	2.2 (3.75)	
Subjects aged 6–12 months		
Breastfed *n* = 29	2.28 (1.41)	0.65
Formula fed *n* = 27	1.77 (1.98)	
Subjects aged 12–18 months		
Breastfed *n* = 38	1.3 (2.5)	0.35
Formula fed *n* = 21	1.8 (1.98)	

a Normally distributed continuous variables were compared with independent sample t-tests. Variables that were not parametrically distributed were analysed with Mann–Whitney U tests.

### Hormone Concentration versus Age

We divided the reference cohort into 3 age groups at enrolment and found that the median (IQR) leptin concentrations were 2.81 (3.49) ng/ml in group 1 (<6 months; *n* = 202), 1.44 (2.27) ng/ml in group 2 (6–12 months; *n* = 59) and 1.77 (2.05) ng/ml in group 3 (12–18 months; *n* = 56). The serum leptin levels were significantly different between groups 1 and 2 (p<0.05) and between groups 1 and 3 (p<0.05).

### External Validation

To externally validate our results, the characteristics and serum leptin levels of the patients in the reference cohort were compared with those of the patients in the validation cohort; no significant differences were found (Table 3).

**Table 3:** Characteristics of the patients in the reference cohort and validation cohort.

Characteristics	Reference Cohort *n = 317*	Validation Cohort *n = 110*	*p*- value^d^ Reference versus Validation cohort
Infant age (days) a	185.09 (160.36)	159.40 (116.18)	0.123
Male Gender b	168 (53%)	50 (45%)	0.248
BMI (kg/m^2^) a	15.71 (2.18)	16.02 (1.98)	0,19
Serum leptin value (total sample) c	2.37 (3.26)	2.78 (3.45)	0.213
Serum leptin value (infants <6 months) c	2.81 (3.49)	3.11 (2.79)	0.27
Serum leptin value (infants 6–12 months) c	1.44 (2.27)	1.8 (1.86)	0.16
Serum leptin value (infants 12–18 months) c	1.77 (2.05)	2.2 (1.5)	0.39

a Mean (standard deviation).

b Number of subjects (%).

c Median (IQR).

d Normally distributed continuous variables were compared with independent sample t-tests. Variables that were not parametrically distributed were analyzed with Mann–Whitney U tests.

### Serum Leptin Reference Values

**Figure 1:**
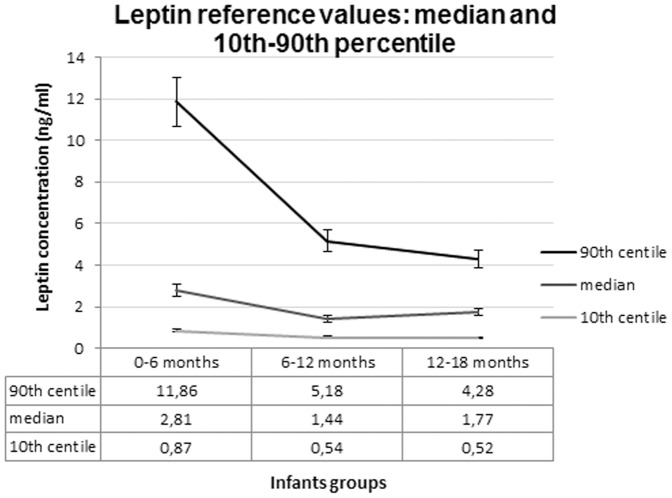
Reference serum leptin concentrations in healthy infants: median and 10^th^–90^th^ centile (Error Bars 10%). (Source: self-elaborated).

We categorized leptin reference values by patient age by calculating the levels of the patients found in the lower and upper percentiles (Figure 1).

### Hormone Concentration versus Gender

We stratified the patients in the reference cohort by gender and found that the median (IQR) leptin concentration was 2.22 (3.11) ng/ml for males (*n* = 168) and 2.6 (3.32) ng/ml for females (*n* = 149); the difference between the two groups was not statistically significant. No significant differences in leptin concentrations were found among the three different age groups (<6 months, 6–12 months and 12–18 months) (Table 2).

### Hormone Concentration versus Type of Feeding

**Figure 2:**
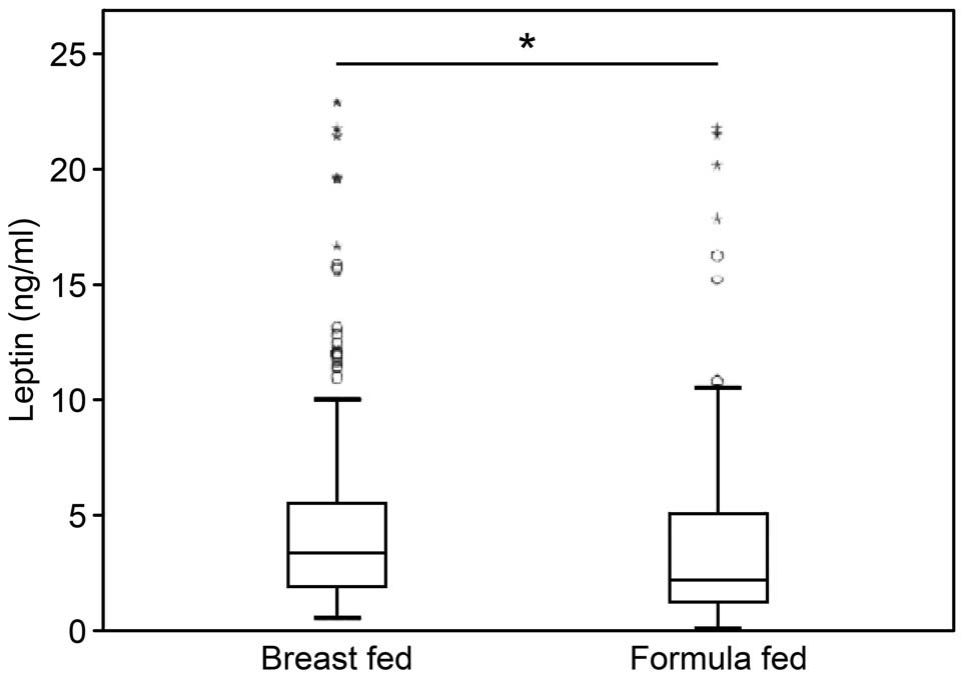
Comparison of serum leptin values in breastfed and formula-fed infants younger than 6 months of age (*n* = 202). The median (IQR) leptin concentration was 3.37 (3.56) ng/ml in the exclusively BF infants (*n* = 121) and 2.2 (3.75) ng/ml in the exclusively FF infants (*n* = 81); the difference between the two groups was statistically significant (* *p*<0.05). (Source: self-elaborated).

Our cohort was divided according to the type of feeding the infants received prior to weaning: the median (IQR) serum leptin concentration was 2.63 (3.34) ng/ml in BF infants (*n* = 188) and 2.12 (2.77) ng/ml in FF infants (*n* = 129); the difference between the BF and FF groups was statistically significant (p<0.05). The median (IQR) leptin concentrations in infants younger than 6 months of age who were not yet weaned (*n* = 202) were 3.37 (3.56) ng/ml (exclusively BF infants (*n* = 121)) and 2.2 (3.75) ng/ml (exclusively FF infants (*n* = 81)); the difference between the two groups was statistically significant (p<0.05) (Figure 2). However, no significant differences in leptin levels were found between the BF and FF infants older than 6 months of age (after weaning) (*n* = 115) (Table 2).

### Hormone Concentration versus BMI

Serum leptin concentrations were not correlated with the BMI of the infant in either the entire sample or in the three different age groups (p>0.05).

## Discussion

In this study, we investigated serum leptin concentrations in a group of healthy infants younger than 18 months of age. We observed that leptin levels decrease when infants begin eating solid foods at 6 months of age. According to our previous studies, BF infants have significantly higher serum leptin levels than FF infants during the first 6 months of life [13], [14]; however, this difference disappears later in life. The reason for this difference is not completely understood. We propose that the leptin found in BF infants includes leptin produced by the adipocytes of the infant and leptin found in human milk. Leptin was detected in human breast milk in 1997 [15], and recent studies have suggested that leptin is destroyed by the pasteurization process used to produce formula [16].

In experimental studies on neonatal rats, Casabiell et al demonstrated that leptin is transferred from the maternal circulation to BM; the leptin present in maternal milk that is ingested during the suckling period can be absorbed by the immature gastric mucosa of the pup and transferred to its circulation [15]. Additionally, leptin receptors have been detected in gastric epithelial cells and in the absorptive cells of the small intestine in mice and humans, which suggests that leptin can pass from milk to infant blood [17]. However, further studies are needed to determine whether leptin present in human milk can exert a biological effect on infants.

Pico et al demonstrated that orally administered leptin is absorbed by the immature gastric epithelium of the neonate and exerts clear biological effects during the early stages of neonatal life, including the down-regulation of endogenous leptin production and the potential control of food intake during this period [18].

Leptin has been proposed to be responsible for some of the beneficial effects of breastfeeding and is thought to be involved in preventing infants to obesity [19]. According to the nutritional programming theory, nutrition during the early period of life is related to the development of childhood obesity, adult obesity and associated pathological conditions. It is well established that genetic, hormonal and perinatal environmental factors acting during the critical period of growth can lead to alterations in the development of organs and systems; in particular, infants who experience alterations in leptin profiles during early life are more likely to develop metabolic disorders later in life. For this reason, this adipocytokine can be considered a potential programming factor [20], [21].

Palou et al. found that supplementing rat pups with oral leptin during the suckling period resulted in a decrease in food intake, a preference for carbohydrates over fat, a reduced likelihood of being overweight in adulthood and improvements in related parameters such as leptin and insulin sensitivity [22]. Therefore, the length of time during which an infant is breast-fed (up to 9 months of age) is inversely related to the infant's risk of being overweight in a dose-dependent manner [23]. In a recent follow-up study, 8-year-old children who were BF as infants had significantly lower weights, BMIs and heights than infants who were FF. In this study, infants with lower serum leptin levels at enrolment had a significantly higher BMI later in life [24].

Our data showed that there were no significant differences in leptin concentrations between males and females in early infancy; however, females tended to have higher serum leptin level than males. These results are in agreement with those of previous studies. Karakosta et al calculated gender-specific reference intervals for leptin levels in cord blood in 398 healthy neonates in Crete and found that females had higher levels of this hormone than males [25]. Leptin levels have been shown to be consistently higher in females than in males later in life [26], [27]. We hypothesize that these differences are related to differences in body composition or hormone levels in males and females.

No significant correlation was found between serum leptin concentrations and the BMI of the infant [8]. This phenomenon could be specific to this period of life, as infants can experience an energy imbalance that is correlated with leptin levels [11].

Leptin reference ranges have been shown to vary significantly, as many parameters such as age, BMI, percent body fat, Tanner pubertal stage, sex, ethnicity, circadian rhythm and assay method can influence the levels of these hormones [28], [29].

To the best of our knowledge, this is the first study to report reference leptin values for a large sample of healthy infants younger than 18 months of age. The advantages of this study included a large sample size and the use of Clinical and Laboratory Standards Institute (CLSI) criteria. Moreover, highly selective criteria (e.g., a limited age range and the exclusion of subjects who were fed both breast milk and formula) were used to enroll participants, and samples were collected under similar laboratory conditions (i.e., in the morning after a three-hour fasting period). RIA methodology, which is used in the majority of published studies on leptin, was used to compare groups in our study.

## Conclusions

A specific reference range of leptin levels can be used clinically to interpret leptin values in healthy infants and in infants with pathological conditions associated with an altered energy balance.

This study presents a range of serum leptin values in healthy infants during the first 18 months of life. We found no differences in leptin levels between males and females during early infancy; however, leptin levels were slightly lower in males greater than 6 months of age. We confirmed that breast-fed infants had significantly higher serum leptin levels than formula-fed infants during the first 6 months of life; later in life, these differences disappeared.

We created and validated a clinically useful tool for identifying infants with higher serum leptin levels.
