# Correction: Serum Reference Values for Leptin in Healthy Infants

**DOI:** 10.1371/journal.pone.0117622

**Published:** 2015-01-09

**Authors:** The PLOS ONE Staff

In the PDF version of this article, the footer erroneously gives the published year and month as “November 2007”. This date in the PDF footer should read “November 2014”.
