# Correction: Serum Reference Values for Leptin in Healthy Infants

**DOI:** 10.1371/journal.pone.0118046

**Published:** 2015-02-06

**Authors:** The PLOS ONE Staff

The PDF file of the published article incorrectly lists the year of publication as 2007, rather than 2014.
